# Crowned dens syndrome—case of crystal deposition in cervical spine

**DOI:** 10.1093/omcr/omab135

**Published:** 2022-01-24

**Authors:** Sabeeh Shams, Behram Khan, Andrew Jeffries

**Affiliations:** Rheumatology Department, Blackpool Teaching Hospital Foundation Trust, Blackpool FY3 8NR, UK; Rheumatology Department, Blackpool Teaching Hospital Foundation Trust, Blackpool FY3 8NR, UK; Rheumatology Department, Blackpool Teaching Hospital Foundation Trust, Blackpool FY3 8NR, UK

## Abstract

Neck pain can be very bothersome for patients, and most will seek analgesic relief as soon as possible. We present a case of crystal deposition in the cervical spine where a frail patient admitted for pneumonia developed severe neck pain raising suspicion of discitis, and did not respond to standard analgesic medications. Investigations revealed crystal deposition around the dens and alar ligaments (C_1_ and C_2_ Spine) suggestive of crowned dens syndrome, which responded promptly to steroid therapy and averting the patient from invasive investigations and intense treatment with antibiotics.

## INTRODUCTION

Crowned dens syndrome (CDS), first described in 1960, usually presents after 60 [1] with neck and shoulder pain. It is a rare metabolic disorder characterized by calcium pyrophosphate (CPP) crystal deposition around the odontoid process and cruciform ligament of C1 and C2 cervical vertebrae [3]. Bloods test usually shows raised inflammatory markers [3, 4, 5], swaying physicians to treat for possible infective causes and perform invasive procedures for neck pain instead of focusing on the benign rheumatological cause, which can be easily managed with conservative management.

## CASE REPORT

We present a case of an independently mobile 90-year-old patient with a background of hypertension, dementia, hypothyroidism, chronic kidney disease stage III, mitral regurgitation, paroxysmal atrial fibrillation, migraines and vitamin D insufficiency, found slumped on a toilet seat and was initially treated for community-acquired pneumonia (CAP–CURB65 = 3), rhabdomyolysis, acute kidney injury and hypoactive delirium. On examination at admission patient was pleasantly confused, had right-sided basal crepitations, no apparent head injury or focal neurological deficit and no cervical spine tenderness and was treated with antibiotics and fluids as per local policy, to which the patient responded well. The patient’s vitals remained stable during the admission.

On Day 7, the patient complained of right-sided neck and shoulder blade pain. C-Spine was tender to touch with a limited range of movement; however, no signs suggestive of meningism were present. Initial analgesic therapy, including paracetamol, codeine, lignocaine patch and oxycodone, had little effect on the patient’s symptoms.

Initial investigations showed raised inflammatory markers, WBC 16.9, CRP 40.9, deranged renal profile with Creatinine 128, Urea 14.4, CK 2480, Lactate 2.4, pH 7.37, HCO3 21.5. For acute neck pain patient had a cervical spine X-ray, which did not show any pathology around the site of tenderness.

**Figure 1:**
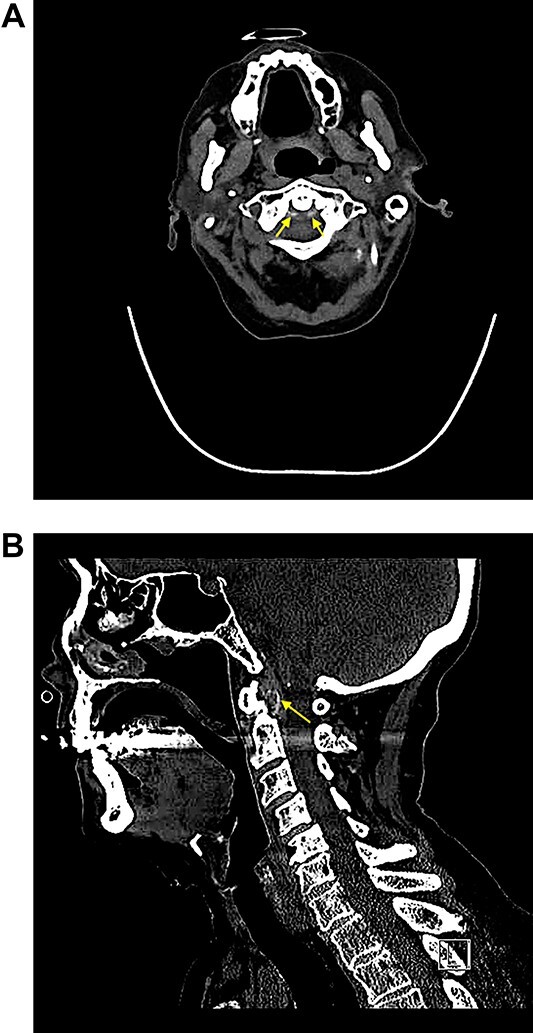
(**a**) Sagittal section of C-Spine showing linear calcium deposition around C1 (Os Dens) and (**b**) Axial section of C-Spine showing linear calcium deposition around C1 (Os Dens,) which revealed thickening + calcification of alar ligaments surrounding dens (yellow arrows) suggesting the diagnosis of crowned dens syndrome.

A computed tomography (CT)-Spine was requested given the inadequate response to standard analgesic therapy [Fig. 1a: Sagittal section of C-Spine showing linear calcium deposition around C1 (Os Dens) and Fig. 1b: Axial section of C-Spine showing linear calcium deposition around C1 (Os Dens)], which revealed thickening + calcification of alar ligaments surrounding dens (yellow arrows) suggesting the diagnosis of crowned dens syndrome.

The rheumatology team reviewed the patient and started on a weaning regimen of prednisolone 30 mg once daily. The patient’s neck pain rapidly improved after initiation with steroids; however, the patient developed hospital-acquired pneumonia, responded poorly to the antibiotics and was fast-track discharged home for palliative care.

## DISCUSSION

CDS, first described in 1960, usually presents after 60 [1] with mild to severe neck and shoulder pain with an occipital headache. Patients often have a fever, worsening neck pain [3, 6] and headache [1] over a short period.

It is more prevalent in females, associated with pseudogout [4], hyperparathyroidism, hemochromatosis, ochronosis, hypophosphatasia [5].

CDS involves CPP or hydroxyapatite crystals deposition around the odontoid process, synovial membrane, articular capsule, transverse ligament, transverse cruciate and alar ligaments [2]. It is usually asymptomatic but can lead to a non-specific inflammatory reaction leading to the attachment of multiple protein molecules on their surfaces. This complex is phagocytosed by neutrophils causing cell damage releasing proteolytic enzymes providing a positive feedback loop, aggravating inflammation [5].

CDS is diagnosed with an unenhanced CT scan of the cervical spine showing thickening of ‘os dens’ [1, 3, 5, 6]; single photon emission computed tomography shows increased uptake at the anterior border of dens [3].

It is crucial to have a high index of suspicion for this relatively benign condition, which can be misdiagnosed as several conditions, including trauma, meningitis, discitis/osteomyelitis, giant cell arteritis, polymyalgia rheumatica, osteomyelitis, pyrexia of unknown origin [3, 4].

Complications may include continuous degeneration and erosion of joints leading to atlantoaxial instability, cord compression and tetra-paresis [3].

Current evidence suggests treating with colchicine [3] or non-steroidal anti-inflammatory drugs (NSAIDs) for 2–3 weeks [5, 6]. If no or inadequate response, then a short course of steroids usually resolves symptoms [3, 5].

CDS should be considered in an elderly patient presenting with new-onset headache, neck pain with raised inflammatory markers, particularly in those with a history of pseudogout, thus avoiding unnecessary invasive investigation like lumbar puncture [3], temporal artery biopsy [4] and treatment with antibiotics and antivirals [3, 5].

## CONFLICT OF INTEREST

Dr Sabeeh Shams, on behalf of all the authors, confirms that we have no conflict of interest.

## FUNDING

No financial support was received to publish this case report.

## ETHICAL APPROVAL

Not required.

## PATIENT CONSENT

We have obtained consent from the patient’s next of kin as the patient has passed away.

## GUARANTOR

Dr Behram Khan.
